# High-throughput drug screening identifies novel therapeutics for Low Grade Serous Ovarian Carcinoma

**DOI:** 10.1038/s41597-024-03869-x

**Published:** 2024-09-19

**Authors:** Kathleen I. Pishas, Karla J. Cowley, Marta Llaurado-Fernandez, Hannah Kim, Jennii Luu, Robert Vary, Nikola A. Bowden, Ian G. Campbell, Mark S. Carey, Kaylene J. Simpson, Dane Cheasley

**Affiliations:** 1 Peter MacCallum Cancer Centre, Melbourne, Victoria 3000 Australia; 2 The Sir Peter MacCallum Department of Oncology, The University of Melbourne, Parkville, Victoria 3010 Australia; 3 Victorian Centre for Functional Genomics, Peter MacCallum Cancer Centre, Melbourne, Victoria 3000 Australia; 4 Division of Gynecology Oncology, Department of Obstetrics and Gynecology, University of British Columbia, Vancouver, British Columbia V5Z 1M9 Canada; 5 Department of Clinical Research, BC Cancer, Vancouver, British Columbia V5Z 4E6 Canada; 6 School of Medicine and Public Health, University of Newcastle, Callaghan, New South Wales Australia; 7 Drug Repurposing and Medicines Research Program, Hunter Medical Research Institute, New Lambton, New South Wales 2305 Australia; 8 Department of Biochemistry and Pharmacology, The University of Melbourne, Parkville, Victoria 3010 Australia

**Keywords:** High-throughput screening, Drug screening

## Abstract

Low grade serous carcinoma (LGSOC) is a rare epithelial ovarian cancer with unique molecular characteristics compared to the more common tubo-ovarian high-grade serous ovarian carcinoma. Pivotal clinical trials guiding the management of epithelial ovarian cancer lack sufficient cases of LGSOC for meaningful subgroup analysis, hence overall findings cannot be extrapolated to rarer chemo-resistant subtypes such as LGSOC. Furthermore, there is a need for more effective therapies for the treatment of relapsed disease, as treatment options are limited. To address this, we conducted the largest quantitative high-throughput drug screening effort (n = 3436 compounds) in 12 patient-derived LGSOC cell lines and one normal ovary cell line to identify unexplored therapeutic avenues. Using a combination of high-throughput robotics, high-content imaging and novel data analysis pipelines, our data set identified 60 high and 19 moderate confidence hits which induced cancer cell specific cytotoxicity at the lowest compound dose assessed (0.1 µM). We also revealed a series of known (*mTOR/PI3K/AKT)* and novel (*EGFR* and *MDM2-p53*) drug classes in which LGSOC cell lines showed demonstrable susceptibility to.

## Background & Summary

Low grade serous ovarian carcinoma (LGSOC) of the ovary or peritoneum is a rare and under-characterised histological subtype of epithelial ovarian tumor which accounts for 6–10% of all serous ovarian tumors and 5–8% of all epithelial ovarian tumors^1^. Compared to the more prevalent high grade serous ovarian carcinoma (HGSOC) subtype, LGSOC’s are often diagnosed at a younger age (47 versus 63 years respectively) and typically display poor overall survival rates due to their late diagnosis and inherently chemo-resistant nature, particularly in the recurrent setting (3–5%)^2–4^. Compared to HGSOC, at the molecular level they also display greater expression of estrogen (ER) and progesterone (PR) receptors and are typically *TP53* wildtype^5–7^.

Due to their rarity, current LGSOC treatments have been broadly established from those based on the genetically highly unstable, *TP53* mutant driven HGSOC. Indeed, it was not until 2004 that LGSOC and HGSOC were recognized as two separate entities based on their genetic landscape, clinical behaviour, disease management and prognosis^8^. As a consequence, primary cytoreductive surgery represents the preferred initial treatment for LGSOC followed by platinum-based chemotherapy^9,10^. Due to their hormone receptor expression, some patients are offered hormone maintenance therapy^5^. The clinical behaviour of LGSOC is remarkably distinct from HGSOC, characterized by low response rates to chemotherapy and a much more indolent course^11,12^. This provides an opportunity and need for effective molecular-based therapies after frontline treatment^5,13^.

Cumulative LGSOC genomic studies have demonstrated a high occurrence of activating mutations in *KRAS, NRAS* and *BRAF* (33–52% cases)^14–16^ underscoring the dominant role that the mitogen activated protein kinase (MAPK) pathway plays in this disease. However, this still leaves a significant proportion of LGSOC’s with unidentified drivers in need of targeted therapeutic strategies. The high prevalence of MAPK pathway alterations in LGSOC and the limited activity of chemotherapy has stimulated the evaluation of selective MEK inhibitors (*selumetinib*, *trametinib*, *binimetinib*), however overall response rates have been underwhelming (15.4–26.6%)^17–19^. Trametinib has been shown to prolong progression-free and overall survival (6–8 months respectively) compared to standard-of-care therapy, however patients are in need of other treatment options after relapse. In addition, no clear consensus exists demonstrating that MEK inhibitors should be limited to a single population based on biomarker stratification (mutational status) as indicated in the National Comprehensive Cancer Network (NCCN) guidelines^11^. Recognizing that LGSOC is a distinct pathologic entity with a different spectrum of underlying molecular genetic events compared to other ovarian cancer histotypes, a unique approach to clinical management is required to maximize patient survival. To address this critical priority, this study employed the largest high-throughput drug screening effort of LGSOC patient-derived cell lines conducted to date to identify novel therapeutic opportunities for these patients.

High-throughput drug screening has revolutionized drug discovery, significantly impacting clinical development through several methods including accelerated identification of potential drug candidates, enhanced success rates, and promotion of a more targeted and efficient approach to drug development^20^. In contrast to traditional high-throughput screening (HTS) methods which typically use a single drug concentration (usually 10 µM), we undertook titration-based quantitative HTS to fully characterize the biological effects of a large and diverse portfolio of candidate compounds (n = 3436) in 12 patient-derived LGSOC cell lines.

Our curated compound collection was sourced from three libraries (FDA/approved, kinase inhibitors and epigenetic targets) for several strategic reasons. Firstly, the FDA/approved library, encompassing all approved and clinically advanced agents (phase I-III), was chosen to expedite movement into signal-seeking studies and reduce the need for time-consuming early-phase trials. This well-curated library provides comprehensive data on targets, signalling pathways affected, mechanisms of action, and drug-drug interactions, allowing us to draw more informed conclusions. Secondly, the kinase inhibitor library was selected as approximately 50% of LGSOC tumors are driven by canonical hotspot MAPK missense mutations, along with rare mutations (eg frameshift mutations in *NF1*) which subsequently lead to MAPK activation. As several single-arm MEK inhibitor clinical trials using common kinase inhibitors have shown poor response rates in LGSOC patients, we therefore aimed to determine if other kinase inhibitors would demonstrate greater efficacy in our LGSOC cell lines. Lastly, the epigenetic library was selected based on LGSOC genomic studies which have revealed genetic alterations in epigenetic regulators (*ASH1L*, *DOT1L*, *ARID1A* etc)^14,21^. Thus, we explored the implications of applying epigenetic therapies to our LGSOC cell lines, potentially uncovering new therapeutic avenues.

Our use of a normal surface epithelium ovary cell line (IOSE-523) as a toxicity control and concentration-response format enabled the comprehensive characterization of the biological effects of test compounds that appeared LGSOC specific. This approach yielded 60 high confidence drug hits that induced cytotoxicity (robust Z-score ≤−2 at 1 µM) in 50% of our LGSOC cell panel with no cytotoxicity observed (robust Z-score ≥−2) in IOSE-523 cells. Together our comprehensive screen serves as a foundation to progress unexplored therapeutic avenues for the treatment of LGSOC.

## Methods

### Cell culture

The IOSE-523 (CVCL_E234) normal ovary epithelial cell line was sourced from the Canadian Ovarian Tissue Bank, University of British Columbia (Vancouver, Canada). The AOCS-2 cell line was established by the Australian and Ovarian Cancer Study Group and provided by Prof David Bowtell (Peter MacCallum Cancer Centre, Australia)^22^. SLC58 cells were kindly provided by Prof John Hooper (Mater Research, Australia). The iOvCa241 cell line was kindly provided by Dr Gabriel E. DiMattia (Department of Oncology, Western University, Canada) at the John and Mary Knight Translational Ovarian Cancer Research Unit.

The remaining LGSOC cell lines were established from patient material obtained through Dr Mark Carey at the University of British Columbia (UBC), includingVOA-10841, VOA-6406, VOA-7681 and VOA-14202. Additional cell lines were derived from the UBC ovarian cancer research program (OvCaRe): VOA-1056, VOA-3448, VOA-3723, VOA-4627, and VOA-4698^23–25^. All cell lines were maintained in M199:MCDB105 media (1:1, (Sigma-Aldrich) supplemented with 10% heat inactivated HyClone fetal bovine serum (FBS) (GE Healthcare Life Sciences) and 1% Penicillin-Streptomycin (Gibco). The same batch of FBS was used throughout the screen (DD19099560). All cell lines were maintained at 37 °C with 5% CO_2_, and routinely tested for mycoplasma (Genotyping Core, Peter MacCallum). Cell line clinical and genomic characteristics are detailed in Table 1.

**Table 1:** Clinical and genomic characteristics of the cell lines used for high-throughput drug screening.

Cell Line	Age at Diagnosis	Pathology at collection	Sample Origin	Treatment status at collection	*BRAF/KRAS/NRAS* mutation status	Seeding density (cells/well)
*iOvCa241*	51	Advanced LGSOC	Ascites	Post chemotherapy	KRAS (G12D)	1800
*VOA-14202*	73	Recurrent LGSOC	Tumor	Post anti-hormone	KRAS (G12V)	1500
*VOA-7681*	59	Advanced LGSOC	Tumor	Treatment naïve	KRAS (G12V)	1700
*VOA-1056*	61	Advanced MPSBT with invasive implants/IIIC	Tumor	Treatment Naïve	NRAS (Q61R)	800
*VOA-6406*	56	Recurrent LGSOC	Tumor	Post chemotherapy	NRAS (Q61R)	1900
*VOA-10841*	72	Advanced LGSOC	Tumor	Treatment Naïve	NRAS (Q61K)	1050
*VOA-3448*	42	Recurrent LGSOC with MP/IC	Ascites	Post chemotherapy and anti-hormone therapy	Wildtype	1000
*VOA-3723*^*a*^	42	Recurrent LGSOC with MP/IC	Ascites	Post chemotherapy and anti-hormone therapy	Wildtype	1900
*VOA-4627*	41	Recurrent LGSOC	Ascites	Post chemotherapy (Carboplatin/Taxol, Gemcitabine), Letrozole, Avastin, and PARP inhibitor	Wildtype	600
*VOA-4698*^*b*^	41	Recurrent LGSOC	Ascites	Post chemotherapy (Carboplatin/Taxol, Gemcitabine), Letrozole, Avastin, and PARP inhibitor	Wildtype	450
*AOCS-2*	28	Recurrent LGSOC	Ascites	Post chemotherapy	Wildtype	750
*SLC58*	67	Recurrent LGSOC	Ascites	Post chemotherapy	Wildtype	1050
*IOSE-523*	Unknown	Ovary, surface epithelium			Wildtype	350

*MP* micropapillary, *MPSBT* micropapillary serous bordeline ovarian tumor, ^a^Matched patient cell line VOA-3448, ^b^Matched patient cell line VOA-4627. Cell seeding density based on 384 well plate format.

### IncuCyte live cell imaging

**Fig. 1:**
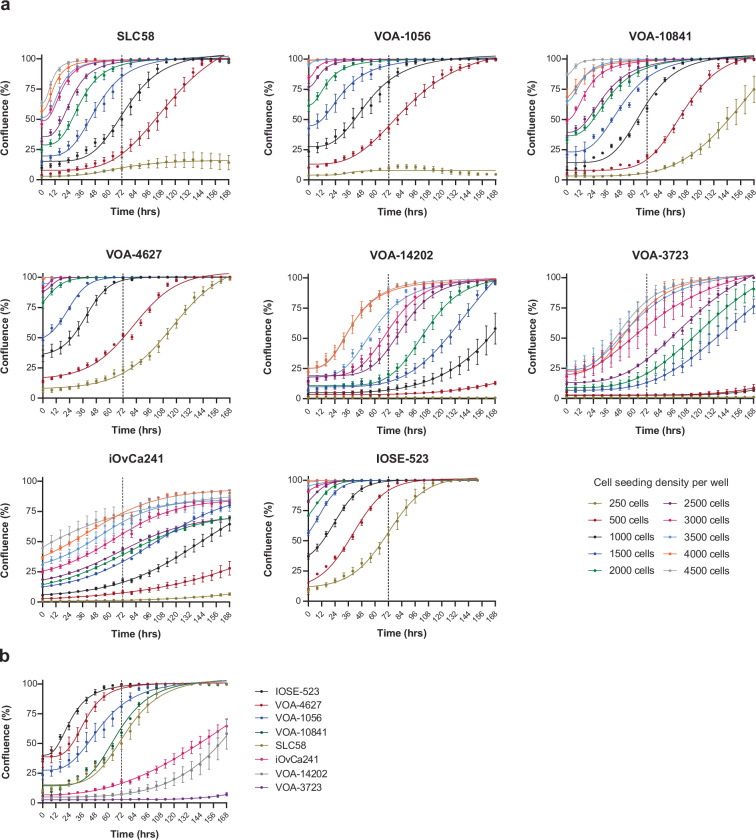
Growth kinetic analysis of cell lines used for high-throughput drug screening. (**a**) Representative IncuCyte confluency kinetics of cell lines. Cell lines were seeded at the indicated concentrations with phase contrast images taken every 8 hr over a 168 hr period. Data represent mean confluency +/− SEM from quadruplicate wells. (**b**) Growth kinetics of 8 cell lines seeded at 1000 cells per well, imaged and analysed as stated previously.

To establish cell line growth kinetics and optimal seeding density, cell lines were seeded (50 µl per well) in black 384 well low flange, clear flat bottom microplates (Corning) and left to adhere overnight. Cells were seeded in 500 cells per well increments (250–4500 cells/well) with quadruplicate wells per seeding density tested. Phase contrast images (10 × magnification) were taken in the IncuCyte SX5 Imaging System (Sartorius, Göttingen, Germany) at 8 hr intervals for a minimum of 168 hr, with media changed every 96 hr. Cell confluence (phase contrast) was evaluated using IncuCyte SX5 software (2022B, Sartorius). For our purposes, a cell was defined as an entity with an area > 450μm^2^. The lowest seeding density required to reach 70–80% confluency 72 hr post scanning was selected for high-throughput drug screening (Fig. 1).

### High-throughput drug screening

**Fig. 2:**
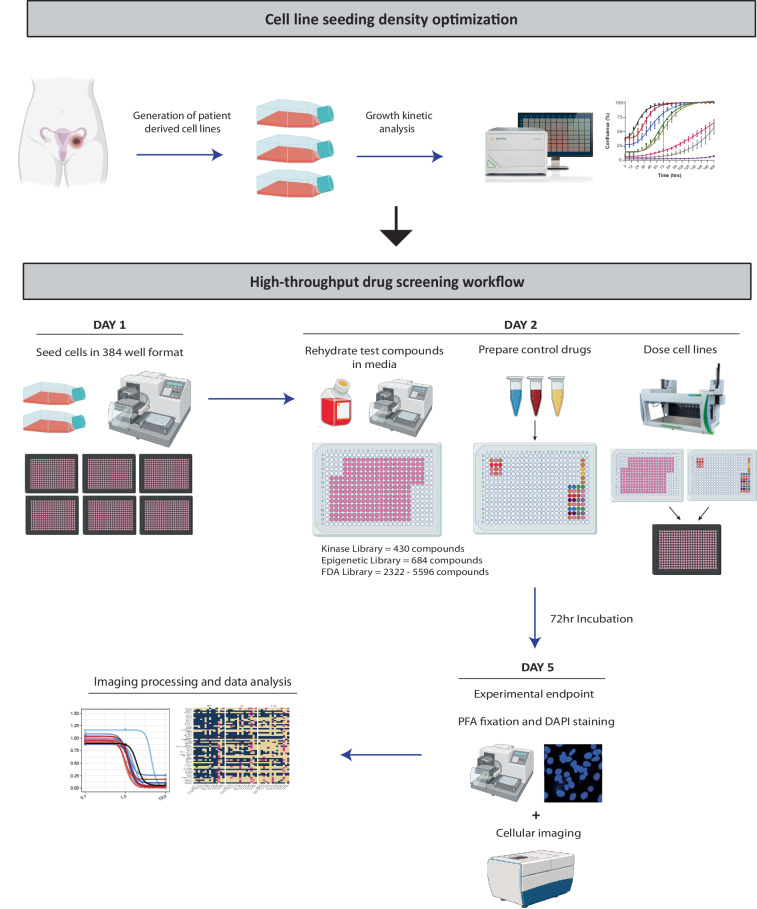
Schematic depicting the workflows for establishing cell line growth kinetics and high-throughput drug screening. Firstly, cell line growth kinetics and optimal seeding density were established through live cell imaging (IncuCyte). For high-throughput drug screening experiments, cell lines were seeded in 384 well format and left to adhere for a minimum of 20 hr. The following day test compounds were rehydrated in cell culture media and control drugs prepared, with all compounds stamped onto cell plates using automated liquid handling platforms. Following 72 hr of incubation cell plates were stained with DAPI to determine viability, and imaged for data processing.

Screen workflow is shown in Fig. 2. Libraries were screened at the Victorian Centre for Functional Genomics (VCFG, Peter MacCallum Cancer Centre, Melbourne, Australia) between the 28^th^ October 2021 - 9^th^ March 2023. Food and Drug Administration (FDA) (n = 5596 compounds), kinase inhibitor (n = 430 compounds), and epigenetic (n = 684 compounds) compound libraries were sourced from Compounds Australia (Griffith University, Australia). Compounds were provided in assay ready (0.1, 1 and 10 µM) dry format and stored at −20 °C until use (on average 1–3 weeks post receipt). The full Compounds Australia FDA library (n = 5596) was screened in 4 cell lines (SLC58, AOCS-2, VOA-6406 and IOSE-523), with the remaining 9 LGSOC cell lines screened with a customized FDA library comprising 2322 compounds we curated to represent Phase 1 - approved agents only. Various negative (media only, 0.2% DMSO) and positive controls (Cisplatin, Carboplatin, Doxorubicin, Mitomycin C, Staurosporine, Paclitaxel; 0.1–10 µM) were included on each 384-well plate to normalise for data collection and reproducibility between plates. The screen and control assay plate layout are shown in Supplementary Fig. 1. Controls were prepared and plated at the VCFG on the same day of screening. Cell seeding, drug plate rehydration and fixing/staining liquid handling steps were performed using a robotic BioTek 406 liquid handling platform (BioTek, Agilent) (dispense Z-height of 336). All fixation and staining solutions were filtered (0.45 µm filter) prior to use. Drug dosing was performed using a JANUS G3 automated workstation (Revvity) with imaging of DAPI nuclear staining performed on a CellInsight CX7 LED high content imager (Thermo Fisher Scientific).

Consumables required for the screen

- 384-well, black walled, tissue culture treated plates, with clear flat bottom wells, sterile, with lid (Corning, Cat #CLS3764)
- V-bottom Polypropylene 384 well microplates (Greiner, Cat #781280)
- Plateloc adhesive aluminium seals (Agilent Technologies, Cat #24210-001)

Cell culture consumables

- M199 Medium (Gibco, Cat #31100-035)
- MCDB 105 Medium (Sigma-Aldrich, Cat # M6395)
- Penicillin-Streptomycin (Gibco, Cat #15070063)
- TrypLE Express Enzyme (1X), no phenol red (Gibco, Cat #12604021)

Fixation and staining reagents

- Dulbecco’s PBS (-CaCl_2_/-MgCl_2_) (Peter MacCallum Cancer Centre Media Kitchen)
- 1 M Tris (HCI) pH 7.4 (Peter MacCallum Cancer Centre Media Kitchen)
- 16% Paraformaldehyde (ProSciTech, Cat #C004) (1:4 dilution)
- 10% Triton-X-100 (Sigma-Aldrich, Cat #X100)
- DAPI (4′,6-Diamidino-2-Phenylindole, Dihydrochloride) (Life Technologies, Cat #D1306); (5 mg/ml, 1:5000).

Control reagents

All control agents except Cisplatin and Carboplatin were diluted in DMSO and stored at a master stock concentration of 50 mM. Cisplatin was diluted in Dimethylformamide (DMF) and Carboplatin was diluted in water.

- Dimethyl sulfoxide (DMSO) Hybri-Max™, sterile-filtered, BioReagent (Sigma-Aldrich, Cat #DS2650)
- Carboplatin (Selleckchem, Cat #S1215)
- Cisplatin (Selleckchem, Cat #S1166)
- Doxorubicin HCI (Selleckchem, Cat #S1208)
- Mitomycin C (Selleckchem, Cat #S8146)
- Paclitaxel (Selleckchem, Cat #S1150)
- Staurosporine (Selleckchem, Cat #S1421)

### Primary and secondary screen workflow

#### Day 1: Cell seeding

Cell lines grown in T175 flask(s) to a maximum of 80% confluency, were prepared for screening by washing twice in PBS followed by incubation in TrypLE Express Enzyme for 2–15 min at 37 °C until detached. The cell pellet was resuspended in growth media and counted (4 tests) using a Countess 3 FL automated cell counter (Thermo Fisher Scientific). Cell suspension was diluted according to optimized seeding density (0.077–0.422 × 10^5^ cells/ml) (Table 1). Cells were robotically seeded (Biotek 406) in columns 1–24 of black walled 384-well tissue culture plates with 45 µl of cell suspension per well (350–1900 cells/well). Plates were briefly centrifuged (1000 rpm for 30 sec) and incubated on a level bench at room temperature for 10 min followed by incubation overnight in a STX200 automated microplate humidified incubator (LiCONiC, 37 °C with 5% CO_2_). Cell lines possessing a long lag phase post trypsinization (VOA-14202, VOA-3723) were seeded 48 hr prior to dosing to ensure cells were in exponential growth phase at the time of drug administration (Fig. 1).

#### Day 2: Drug treatment

Compound source master plates were warmed to room temperature for 15 min, briefly centrifuged (1000 rpm for 30 sec) and rehydrated (BioTek 406) with pre-warmed growth media based on compound concentration. Master plate layout is depicted in Supplementary Fig. 1a. Master plates were sealed, briefly centrifuged (1000 rpm for 30 sec) and incubated (37 °C with 5% CO_2_) prior to drug dosing. Master plates were stamped (5 µl per well) to experimental plates using a JANUS G3 automated workstation.

For secondary screens, compounds (n = 79) purchased from MedChem Express were dissolved to 10 mM working stock aliquots in DMSO. Each compound was prepared as a 10X concentrated (100 µM) stock in media and hand-pipetted into 384 well control plates. Compounds were then serially diluted to 1 and 0.1 µM using the Sciclone ALH 3000 Workstation (Caliper Life Sciences) and stamped (5 µl per well) to experimental plates using the ALH 3000. Technical replicates were performed across two independent experimental plates.

10X concentrated control compounds (Cisplatin, Carboplatin, Doxorubicin, Mitomycin C, Paclitaxel, Staurosporine) (final concentration 0.1 and 10 µM) were prepared fresh on the day of drug dosing and aliquoted into 384 well V-bottom plates as per the control plate layout (Supplementary Fig. 1b) with media and DMSO controls. Compounds were stamped (5 µl per well) as described above. Following drug dosing all plates were placed in a LiCONiC STX200 automated microplate humidified incubator (37 °C with 5% CO_2_) and incubated for 72 hr.

#### Day 5: Fixation and staining

Following 72 hr of compound treatment, growth media was aspirated and replaced with 25 µl of fixative (4% PFA in PBS) (BioTek 406). All aspiration steps were performed using a Z-height of 40 (5.08 mm) above carrier, leaving 10 µl residual volume to ensure that the cell monolayer was not disturbed. Plates were incubated for 10 min at room temperature followed by aspiration of fixative and PBS wash (50 µl). Cells were then incubated with 25 µl of staining solution (1 µg/ml DAPI, 2% Triton-X-100, 5% TRIS-HCI in PBS) for 20 min protected from light (aluminium foil) at room temperature. Fixative was then aspirated and cells were washed twice in 50 µl of PBS. For imaging, plates were sealed using the plateloc aluminium seals and then briefly centrifuged (1000 rpm, 30 sec).

### Refinement of drug libraries

Initial pilot screening was conducted across three LGSOC (SLC58, AOCS-2, VOA-6406) and one control (IOSE-523) cell line encompassing a comprehensive collection of 6710 agents across three compound libraries. This extensive collection was then narrowed down to 3436 compounds that were used in all subsequent primary screens across an additional nine LGSOC cell lines. Compounds were removed based on their clinical indication categorization of “investigative” as detailed in the Therapeutic Target^26^ and Drugbank 6.0^27^ databases. Investigative agents were removed to align with the study’s overarching objective to expeditiously advance compound discoveries into practical applications for LGSOC patients. As such our final compound collection focused on agents marked as preclinical or in various phases of clinical development (phase I-III and approved) (n = 3436 agents).

### Imaging

DAPI-stained plates were imaged on the CellInsight CX7 LED high content imager in widefield mode using the Blue excitation LED (386/23 nm) and BGRFRN emission filter. Images were acquired at 10X magnification and 2 × 2 binning (9 fields/well comprising the entire area of the well). Exposure times were calculated to an image saturation target of 35%.

### Image analysis

Images were analysed for cell counts using the open-source modular image analysis software CellProfiler (version 4.1.3)^28^. Nuclear identification and segmentation settings were customised for each cell line. All image analysis pipelines are available in the BioImage Archive (http://www.ebi.ac.uk/bioimage-archive) under the accession number S-BIAD1069.

### Data analysis

Data analysis was performed in R Studio using R version 4.3.1. The R project file and git repository was created with the workflowr package (version 1.7.1) for reproducibility and version control.

#### Normalisation

The raw cell counts (total number of DAPI-stained nuclei identified in each well) were corrected for potential plate location or edge effects using the Loess-fit smoothing method^29^. To prevent highly toxic compounds from being mistaken for plate location effects, wells with cell counts <50% of the negative controls (0.2% DMSO and media) were excluded prior to smoothing and then added back into the dataset after smoothing was complete. Smoothed cell counts (Count_Cells_fit) were then normalised for batch effects by fold changing to the median of the 0.2% DMSO control wells on a per-plate basis.

#### Cell viability binning

Compounds were categorised into the following viability bins based on the normalised cell count: high viability (>1.15), normal viability (0.8 - 1.15), moderate viability (0.5 - 0.8) and low viability (<0.5).

#### Robust Z-scoring

The 10 µM compound concentration was found to be extremely toxic in the majority of compounds and was therefore removed from further analysis due to non-specific toxicity. The normalised cell counts (Count_Cells_Norm_fit) for the refined compound list at 0.1 µM and 1 µM were robust Z-scored using the following formula. Each cell line was calculated separately.

Robust Z-score = (sample value - median of all sample values)/median absolute deviation of all sample values

#### Identification of hit compounds

**Fig. 3:**
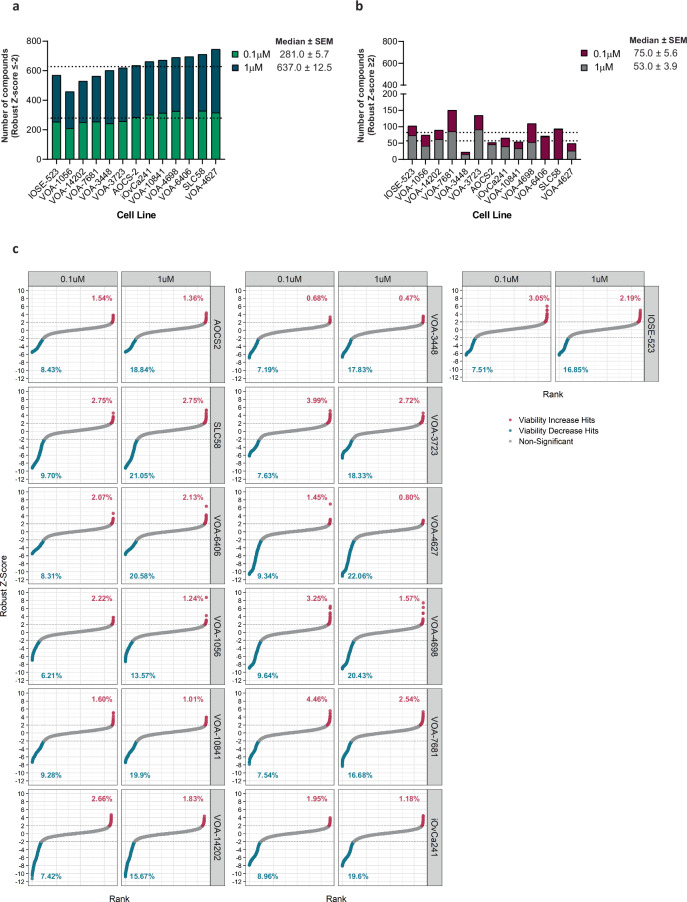
Screen outcomes across all 13 cell lines utilised in this study. (**a**) Number of compounds that induced cytotoxicity (robust Z-score ≤ −2) or significantly increased proliferation (robust Z-score ≥ 2). Dashed line represents median number of hits as per the indicated concentration (0.1 µM and 1 µM). (**b**) Waterfall plots demonstrating the proportion of compounds designated as significant by a robust Z-score cut off ≤ −2 or ≥ 2 (indicated by dashed lines) across the cell line panel at 0.1 µM and 1 µM. The percentages are calculated based on the number of compounds in the primary screen library.

To identify agents that could be used therapeutically for the treatment of LGSOC, compounds were filtered using a robust Z-score cutoff of ≤−2 as an indicator of cytotoxicity (approximately 50% cell death). Using this cutoff, the mean number of compound hits across all cell lines was 281 (range 210–328) at 0.1 µM and 637 (range 459.0–746.0) at 1 µM (Fig. 3a). At the lowest dose tested (0.1 µM), the highest number of hits were observed in SLC58 cells (9.70%) and the lowest number of hits in VOA-1056 cells (6.21%) (Fig. 3c). Conversely, a smaller proportion of compounds were observed to induce a significant increase in cell proliferation (robust Z-score ≥ 2). The mean number of compounds that resulted in increased proliferation across all cell lines was 75 (range 23–151) at 0.1 µM and 53 compounds (range 16–93) at 1 µM (Fig. 3b). No significant difference was observed when comparing hit rates between LGSOC cell lines and our control ovarian surface epithelial cell line (IOSE-523) (Fig. 3).

#### Selection of hits for secondary validation

**Fig. 4:**
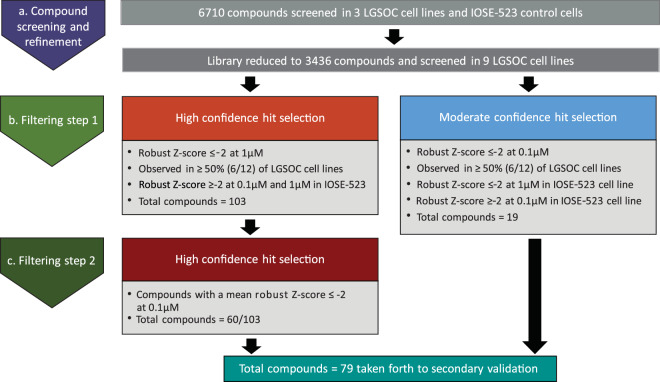
Hit selection strategy applied to the primary screen. (**a**) A total of 6710 compounds were screened and viability assessed across 4 cell lines. Based on this data, we subsequently reduced the library scale by including only agents in phase 1 development trials through to FDA approved and kinase and epigenetic. (**b**) Putative hits were triaged in the initial filtering step based on high and moderate-confidence filtering strategies, resulting in 122 compound hits. (**c**) High confidence hits were further selected for compounds demonstrating cytotoxicity (robust Z-score ≤ -2) at 0.1 µM.

**Fig. 5:**
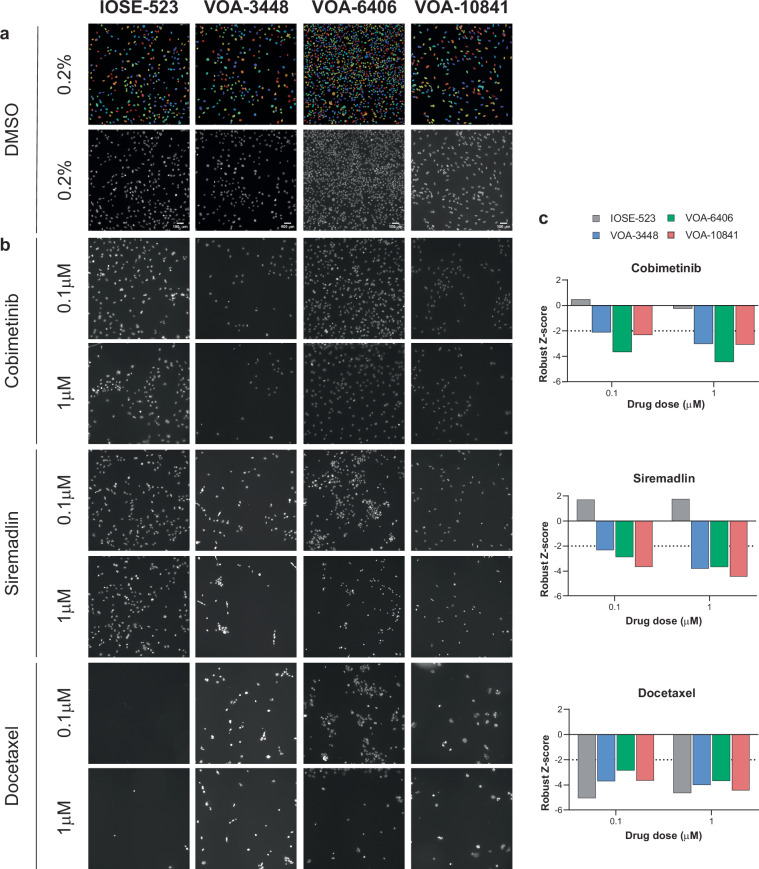
Cell line compound sensitivity. Representative CellProfiler segmentation (top) and brightfield DAPI images of IOSE-523 (toxicity control) and LGSOC cell lines (VOA-3448, VOA-6406, VOA-10841) treated with (**a**) 0.2% DMSO (vehicle control) or (**b**) Cobimetinib, Siremadlin and Doecetaxel for 72hrs (0.1 and 1 μM). Images taken at 10X magnification. (**c**) Robust Z-score values for compounds depicted in (**b**), with values < −2 indicating cytotoxicity.

A systematic tiered approach was utilised to guide selection of cytotoxic compounds for secondary validation (Fig. 4). This strategy involved two sequential steps, which categorized compounds into high or moderate-confidence hits. High-confidence hits were selected based on cell viability (robust Z-score ≤−2) at a clinically relevant dose (1 µM) (Fig. 4b). For inclusion, hits needed to be observed in ≥ 50% of all LGSOC cell lines and have a robust Z-score ≥ 2 (no cytotoxicity) in the control IOSE-523 cell line, thus strengthening the selection that our compound hits are likely to be LGSOC-specific. For example, although the anti-microtuble inhibitor Docetaxel was identified as a compound hit in 100% of our LGSOC cell line panel (average robust Z-score −4.753 ± 0.324, 1 μM), severe toxicity was also observed in IOSE-523 control cells (robust Z-score −4.634, 1 μM) thereby precluding this compound for further investigation (Fig. 5). In contrast, both Cobimetinib (MEK1/2 inhibitor) and Siremadlin (HDM2 inhibitor) were identified as hits (robust Z-score < −2, 1 μM) in 100% and 75% of cell lines respectively with no effect on IOSE-523 cells (robust Z-score -0.249 and 1.758, 1 μM) respectively.

The decision to employ an additional ‘moderate confidence hit selection’ (Fig. 4b) was prompted by the acknowledgment that the high confidence hit selection might have limitations, particularly when relying on the response of a single control cell line. Unlike the high-confidence hit selection, the criteria for moderate-confidence hits was less stringent, recognizing the complexities of compound responses and attempting to capture a broader range of potentially impactful hits for further consideration and validation. For moderate confidence hits, selection stringency was relaxed to include compounds that exhibited an effect at 0.1 µM in ≥ 50% LGSOC of all LGSOC cell lines, and a significant robust Z-score of ≤−2 at the higher clinically relevant dose (1 µM) in the IOSE-523 cell line. The moderate confidence hit selection aims to strike a balance between sensitivity (capturing more potential hits) and specificity (mitigating compounds that target ‘normal’ tissues). The approach acknowledges that certain compounds may demonstrate their efficacy at a higher dose, and this should not necessarily lead to exclusion. In total, this tiered approach successfully identified 60 high-confidence and 19 moderate-confidence hits, demonstrating its effectiveness in refining the selection process for cytotoxic compounds.

For high confidence hits, a final filtering step was employed to only advance compounds displaying a mean robust Z-score of ≤−2 at 0.1 µM across the cell lines (Fig. 4c). This ultimate low-dose step aimed to include compounds with the highest likelihood of effectiveness at physiologically achievable doses, mitigating off-target effects, and narrowing the therapeutic window for subsequent pre-clinical studies. In all 79 compounds were selected for secondary validation studies (Supplementary Table 1).

##### Secondary validation

Compounds selected for secondary validation (n = 79) were independently sourced (MedChem Express) and tested at 3 doses (0.1, 1 and 10 µM) across 4 cell lines (VOA-4698, VOA-3448, VOA-10841 and iOvCa241) to assess reproducibility of compound sensitivity observed from primary screening. Of these, 15 compounds failed (19.0%) to reproduce primary screening results (Supplementary Fig. 2) as defined by a minimum of 50% cell death at 10 µM not achieved in a minimum of 3 of the 4 cell lines. These failed compounds were from several targets including histamine receptor agonists (Decloxizine, Desloratadine), anti-bacterial (AFN-1252) and elastase inhibitors (BAY-85-8501).

## Data Records

### Data record 1

Fluorescent images of DAPI-stained nuclei acquired at 10X magnification for both the primary and secondary screens are publicly available in the BioImage Archive (http://www.ebi.ac.uk/bioimage-archive) under the accession number S-BIAD1069^30^.

The images are provided, as acquired and analysed, in their raw file format (C01 file type, greyscale, 9 fields/well) and are labelled using the nomenclature: MFGTMPcx7_231007040001_A01f00d0. This naming convention reflects: Instrument (MFGTMPcx7), VCFG internal unique plate identifier (231007040001), well ID (A01), field number in the well (f00) and channel (d0). Plate and well annotations, such as cell line, compound name and concentration, are included in the repository as image metadata.

### Data record 2

Cell viability data for the entire primary screen at all concentrations tested are available as comma separated values (CSV) file type in the BioImage Archive under the accession number S-BIAD1069^30^. The file is labelled Pishas_et_al_2023_Data_Record_2 and includes both the comprehensive compound list screened in the first four cell lines and refined compound list screened in subsequent cell lines. Raw, smoothed and normalised (fold change to DMSO median) cell counts and cell viability bin results are provided for every well. The values are annotated with the cell line, compound library, vendor compound ID (QCL_Sample_Number), compound name, concentration, pathway and target.

### Data record 3

Hit selection data for the primary screen refined compound list only at 0.1 µM and 1 µM concentration are available as CSV file type in the BioImage Archive under the accession number S-BIAD1069^30^. The file is labelled Pishas_et_al_2023_Data_Record_3. Raw, smoothed and normalised (fold change to DMSO median) cell counts, cell viability bin results and robust Z-scores are provided for every well. The values are annotated as described in Data Record 2.

### Data record 4

Cell viability data for the entire secondary screen at all concentrations tested are available as CSV file type in the BioImage Archive under the accession number S-BIAD1069^30^. The file is labelled Pishas_et_al_2023_Data_Record_4. Raw, smoothed and normalised cell counts are provided for every well. The values are annotated as described in Data Record 2.

## Technical Validation

To ensure the robustness and reproducibility of the high-throughput drug screening approach, several quality control procedures were implemented and assessed.

Experimental design: To assess the level of variability within each plate and across all plates in the screen, the experimental design included multiple well replicates of DMSO and media only negative controls (14 and 12 wells, respectively) per plate and duplicate wells of each of the positive controls (Cisplatin, Paclitaxel, Doxorubicin Mitomycin C and Staurosporine). This was essential to validate the integrity and reliability of the data generated over the many months of screening. A single well replicate per plate was included for the positive control Cisplatin due to space constraints, however multiple replicates of Carboplatin were assessed due to its clinical relevance to LGSOC (standard of care agent).

**Fig. 6:**
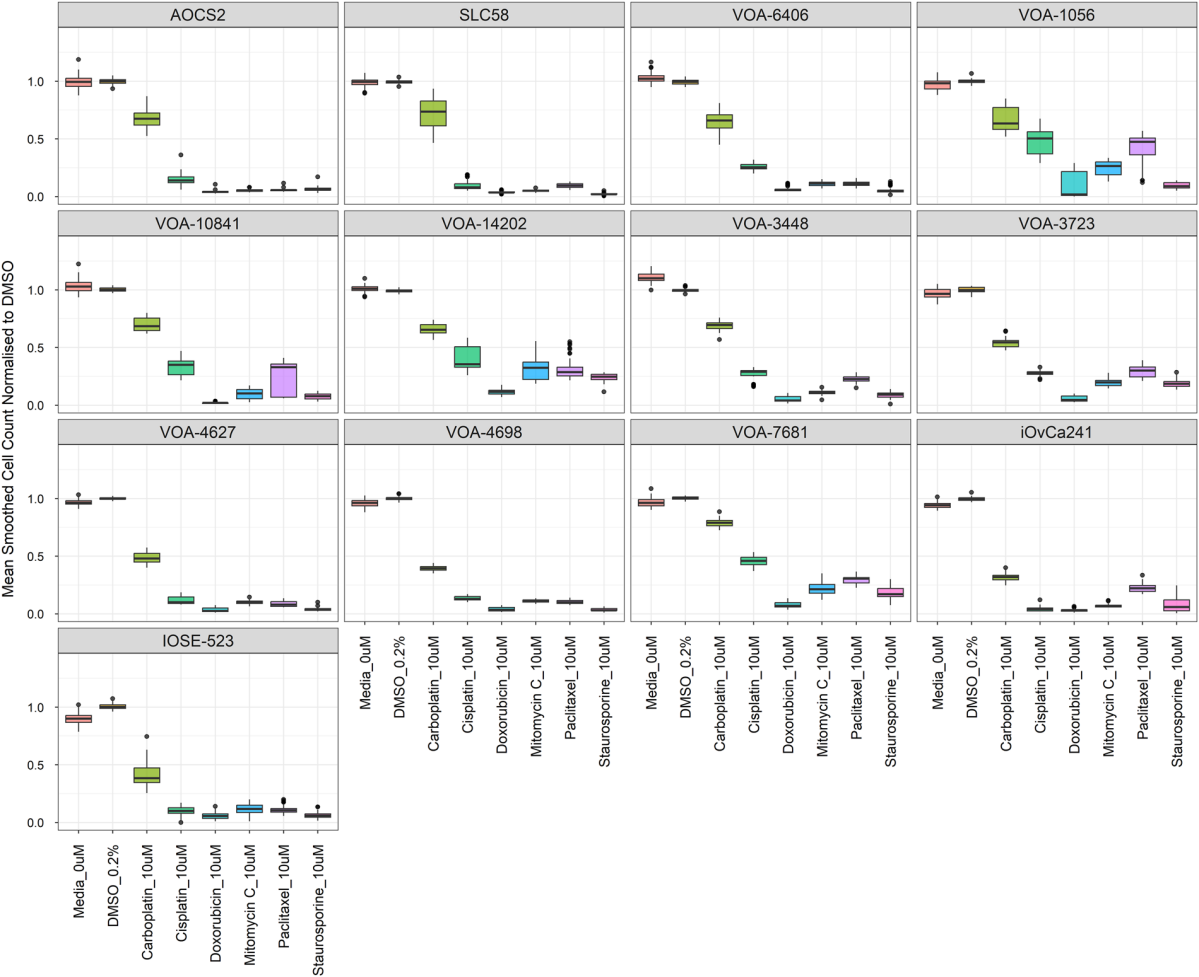
Quality metrics of positive and negative controls across the primary screen for all cell lines. Positive control compounds (Carboplatin, Cisplatin, Doxorubicin, Mitomycin C, Paclitaxel, and Staurosporine) were screened at 10 µM (duplicate wells per plate) and were normalised to 0.2% DMSO control wells. Box and whisker plots show the distribution of the normalised mean per plate cell counts. Outliers are defined as having values > 1.5 × IQR (interquartile range) outside of the 25th to 75th percentile range and are represented in the plot by black dots. Untreated, media only negative controls were also normalised to 0.2% DMSO.

Screen quality assessment: The quality of the screen was primarily evaluated through the performance and reproducibility of both negative (media only and DMSO) and positive (Carboplatin, Cisplatin, Paclitaxel, Doxorubicin, Mitomycin C and Staurosporine) controls across all plates and cell lines (Fig. 6). Key metrics for this evaluation were calculated using the cell count normalised to DMSO for each compound (Supplementary Table 2).

### Control Performance

- Negative controls: The mean cell counts normalised to DMSO across all cell lines ranged from 0.99–1.01 for DMSO and 0.90–1.11 for media only controls, indicating high consistency and a lack of toxicity resulting from 0.2% DMSO alone.
- Positive controls: The level of toxicity resulting from positive controls was consistent across all plates for each cell line. Staurosporine was the most toxic positive control in all cell lines, with mean cell counts normalised to DMSO ranging from 0.02–0.24. Carboplatin was the least toxic positive control in all cell lines, as expected, but cell counts normalised to DMSO varied across cell lines, ranging from 0.32–0.79.
- Z’ Factor: The dynamic range between positive controls and DMSO on each plate was assessed using the Z’ Factor. Failing Z’ Factor values (<0) were observed for Carboplatin in 9/12 cell lines screened, which was expected given the consistently low level of toxicity of Carboplatin in these cell lines. Robust Z’ Factor values were achieved for all other positive controls on all plates in the screen. Across cell lines, mean Z’ Factor values ranged from 0.54–0.78 for Doxorubicin, 0.40–0.73 for Mitomycin C, 0.20–0.77 for Paclitaxel and 0.39–0.77 for Staurosporine. The positive controls were most toxic overall and had higher Z’ Factor values in the cell lines VOA-4627 and SLC58 and were the least toxic with lower Z’ Factor values in the cell lines VOA-3723 and VOA-1056.

### Control Reproducibility

- Negative controls: The mean percent coefficient of variation (%CV) across all plates ranged from 5.62–13.31% for media only and 6.21–13.26% for DMSO controls in the cell lines screened. The low %CV for both negative controls in all cell lines indicates minimal variability and high consistency in the baseline measurements, confirming the stability of the screening conditions.
- Positive controls: The positive controls exhibited the following mean %CVs across all plates for all cell lines:
  - Carboplatin: 4.26–14.08%
  - Cisplatin: 5.17–18.63%
  - Doxorubicin: 15.80–53.97%
  - Mitomycin C: 7.98–26.87%
  - Paclitaxel: 5.00–16.21%
  - Staurosporine: 15.02–40.29%

The higher %CV observed for Doxorubicin, Mitomycin C and Staurosporine is attributed to the extreme toxicity of these compounds, which inherently leads to greater variability. However, the consistent levels of toxicity, reported as cell counts normalised to DMSO, observed across all plates confirm the reproducibility of these results. In summary, the comprehensive quality control measures, including low %CVs for negative controls, consistent toxicity levels for positive controls, and robust Z’-factor values for all positive controls except for Carboplatin, all indicate a highly reliable and reproducible screening campaign.

## Usage Notes

Women diagnosed with LGSOC are in desperate need of new therapies specifically tailored towards the unique molecular characteristics of this rare disease entity. To address this unmet need, we undertook the largest quantitative high-throughput drug screening effort (maximum of 6710 compounds) to date to identify novel LGSOC treatment strategies using an expansive panel of 12 well molecularly characterized patient derived LGSOC cell lines. We utilised a stringent tiered approach to hit selection and triaged compounds based on their cytotoxicity against a normal ovarian surface epithelium cell line (IOSE-523). Using a robust Z-score cutoff of ≤−2, without filtering 8.3% (0.1 µM) and 18.7% (1 µM) of screened compounds were identified as hits across our LGSOC cell line panel.

Traditional small scale drug screening methods often focus on specific targets or pathways which may overlook potential therapeutic opportunities. For example, Gray *et al*., assessed 42 small molecule drugs in one LGSOC patient organoid sample which were selected based on standard-of-care chemotherapies, and drugs targeting common cancer genes and pathways in cancer/LGSOC^31^. Similarly, Murumägi *et al*., screened 526 approved and investigational compounds across 7 LGSOC patient derived cell lines generated from five patient samples^32^. Our methodology for assessing a broad spectrum of compounds, targeting diverse and well-established biological pathways, fosters innovation by questioning prevailing treatment paradigms in ovarian cancer. This approach enables the identification of unconventional drug candidates with the potential to revolutionize LGSOC patient care. In addition, the inclusion of multiple compound doses instead of the traditional single compound concentration also permitted the selection of hits within physiologically achievable ranges. This will be essential for future synergistic studies evaluating the safety, tolerance, dependence, drug interactions and long-term therapeutic effects of our lead compounds.

**Fig. 7:**
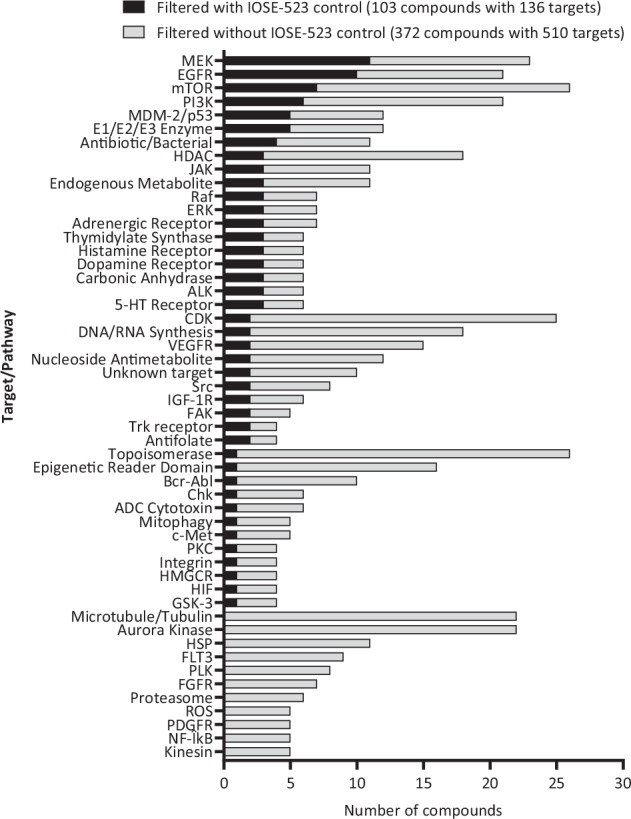
Comparison of target/pathways associated with high confidence hits in LGSOC cell lines. Black and grey bars indicate with and without filtering against the ovarian surface epithelium control (IOSE-523) cell line respectively. Only targets/pathways identified at least twice are shown.

The inclusion of a normal ovarian control cell line was a crucial step in our LGSOC drug screening strategy and permitted i) a baseline for cytotoxic comparison and ii) assessment of the on-target effects of compounds on cancerous versus normal cells. Our specific LGSOC cancer cell cytotoxicity approach filtered out several historical chemotherapeutic compounds including 5-FU, doxorubicin, docetaxel, cyclophosphamide, and chlorambucil, which are often associated with severe dose limiting side effects. This is clearly demonstrated in the target/pathway classes of compound hits identified with and without the control cell line, where we now deliberately excluded the IOSE-523 control filter (robust Z-score ≥ -2 in IOSE-523 cell line) from our high confidence hit selection filtering step in Fig. 7.

Filtering of our high-confidence hits (robust Z-score ≤ -2 at 1 µM) (Fig. 4b) against the IOSE-523 control, resulted in the identification of 103 compounds which were associated with 136 different molecular targets/pathways (Fig. 7, Supplementary Table 3). Of these the *MEK/RAF/ERK* and *mTOR/PI3K/AKT* signalling pathways were highly enriched (Fig. 7). This is promising given that hotspot mutations in *KRAS, NRAS* and *BRAF* represent the largest molecular subtype of LGSOC^14^. In addition, cytotoxicity to EGFR inhibitors was also observed and expected given that previous *in vitro* studies have shown synergistic efficacy between EGFR (*Erlotinib*) and MEK inhibitors (*Trametinib, Selumetinib*) in MEK resistant LGSOC cell lines^23^.

Conversely, if we excluded the IOSE-523 filter from our high-confidence filtering strategy, 372 compounds associated with 510 molecular targets/pathways would have proceeded to secondary validation (Fig. 7, Supplementary Table 3). This marks a substantial 261% increase in hit compounds (103 versus 372) and a 275% increase in compound-matched targets (136 targets versus 510) when comparing the control inclusion versus control exclusion filtering steps, respectively. Critically, the absence of the IOSE-523 control filter results in a heightened proportion of compounds targeting traditional chemotherapy pathways such as topoisomerase, and microtubule/tubulin inhibitors, along with CDK and aurora kinase inhibitors which have been shown to be ineffective in several preclinical/clinical HGSOC and LGSOC studies^33–35^. This was demonstrated in a recent LGSOC drug screen lacking a normal control cell line which identified 16 compound hits as defined by a 50% reduction in nuclei in > 3 cell lines at 1 μM dose, that appeared to have no biological relevance to the genomic or molecular biology of LGSOC (anti-parasitic, nuclear export, proteasome, HDAC inhibitors, histamine receptor, anti-depressant)^36,37^. Importantly of these 16 agents, 8 were also tested in our screen in which 87.5% (7/8) induced significant non-cancer specific toxicity as observed in our IOSE-523 control cell line (robust Z-score < −2) and were hence subsequently ruled out for further analysis (Abemaciclib, Dinaciclib, Ixazomib, Dasatinib, Homo-harringtonine, Panobinostat, and Volasertib). The non-specific cancer activity of these identified compounds could potentially limit their clinical feasibility due to off target effects.

The three major strengths of our study include inclusion of i) a large collection of patient derived LGSOC cell lines (n = 12), ii) a comprehensive library of compounds tested at varying concentrations which were sourced using an unbiased approach and from a wide mechanism of action range and iii) the use of a normal cell line to serve as a toxicity reference. Screening of predominantly FDA approved and early investigation (Phase I-III) compounds with established maximum tolerated dose, pharmacokinetic and pharmacodynamic profiles potentially enables these candidates to be repurposed for the treatment of other indications such as LGSOC. It also bypasses the need to conduct expensive and lengthy maximum tolerated dose, pharmacokinetic and pharmacodynamic studies required for compounds without known targets or bioavailability profiles. It must be noted that due to limited LGSOC cell line resources, our cell line panel was unable to capture all LGSOC molecular subtypes, predominantly MAPK/USP9X mutant and no specific molecular profile origin, which could limit the applicability of our findings to rarer forms of LGSOC.

In summary, our meticulous and robust screening approach not only contributes to the development of more effective and tailored therapies for LGSOC but also minimizes the risk of potential adverse effects on healthy tissues. Our findings emphasise the necessity for personalized and precise treatment strategies tailored directed towards the basic biology of rare cancers instead of extrapolating clinical findings from more prominent ovarian cancer subtypes. Importantly, our data set serves as an exemplary LGSOC compound sensitivity resource for all cancer researchers to mine.

## Supplementary information

Supplementary Figures

Supplementary Tables 1-3

## Data Availability

The R code used to analyse the data is publicly available in the following BitBucket repository: https://atlassian.petermac.org.au/bitbucket/projects/VCFG/repos/pishas_et_al_2024_ovarian_compound_screen.
