# Supplementary material for: High-throughput drug screening identifies novel therapeutics for Low Grade Serous Ovarian Carcinoma
Source: Sci Data. 2024 Sep 19;11:1024. doi: 10.1038/s41597-024-03869-x (PMC11413243; doi:10.1038/s41597-024-03869-x)
Supplement: Supplementary file 1 — Supplementary Figures [file 41597_2024_3869_MOESM1_ESM.pdf]

**Supplementary Figure Table of Contents**

High-throughput drug screening identifies novel therapeutic avenues for the treatment of Low Grade Serous Ovarian Carcinoma


| Figure | Page Number |
| --- | --- |
| Figure S1. Test Compound Layout | 2 |
| Figure S2. Secondary Validation Dose Response Curves | 3 |

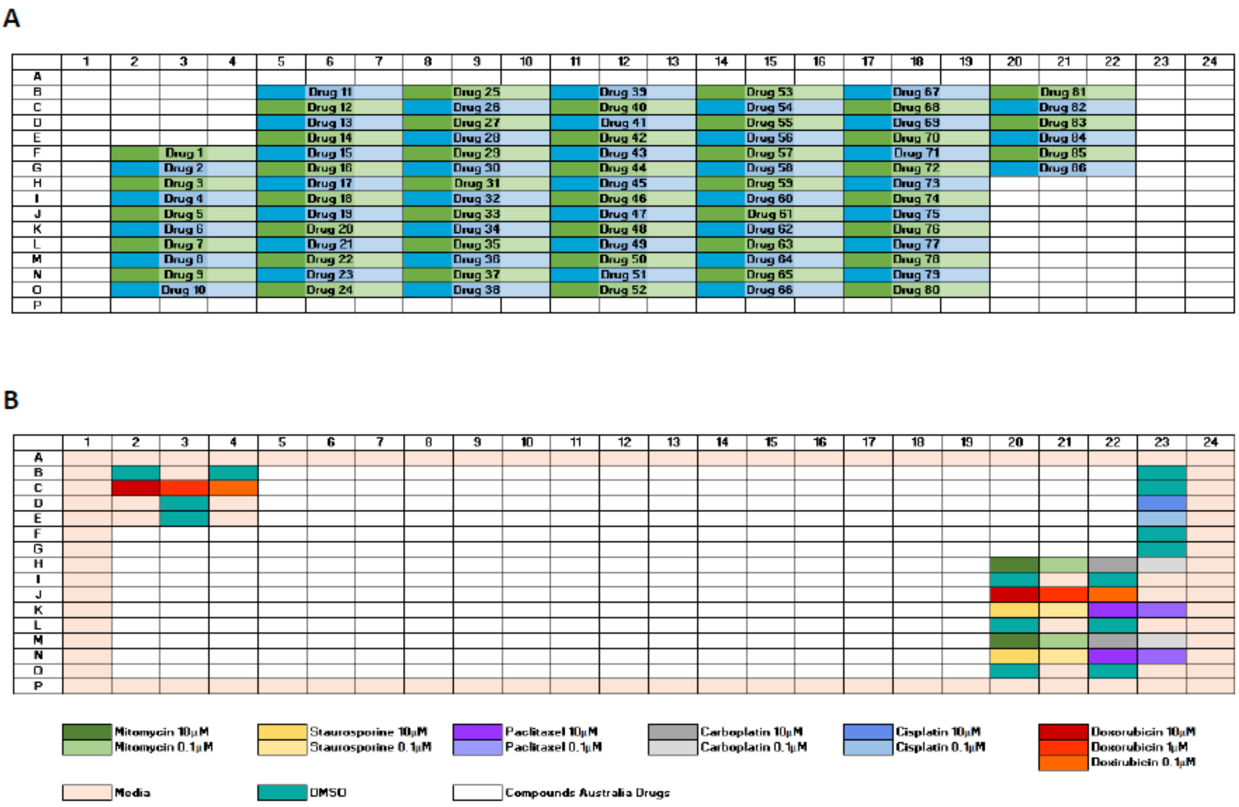


**Supplementary Fig. S1:** Test compound and control drug layout. (A) Compounds Australia drug plate layout. All compounds (86 per plate) were tested at a final concentration of 10, 1 and 0.1µM. (B) Positive (Carboplatin, Cisplatin, Doxorubicin, Mitomycin, Paclitaxel, Staurosporine) and negative (Media only, 0.2% final concentration DMSO) control compound location. Final test concentration indicated. Both Compounds Australia and control agents were plated in 384 well plate format.

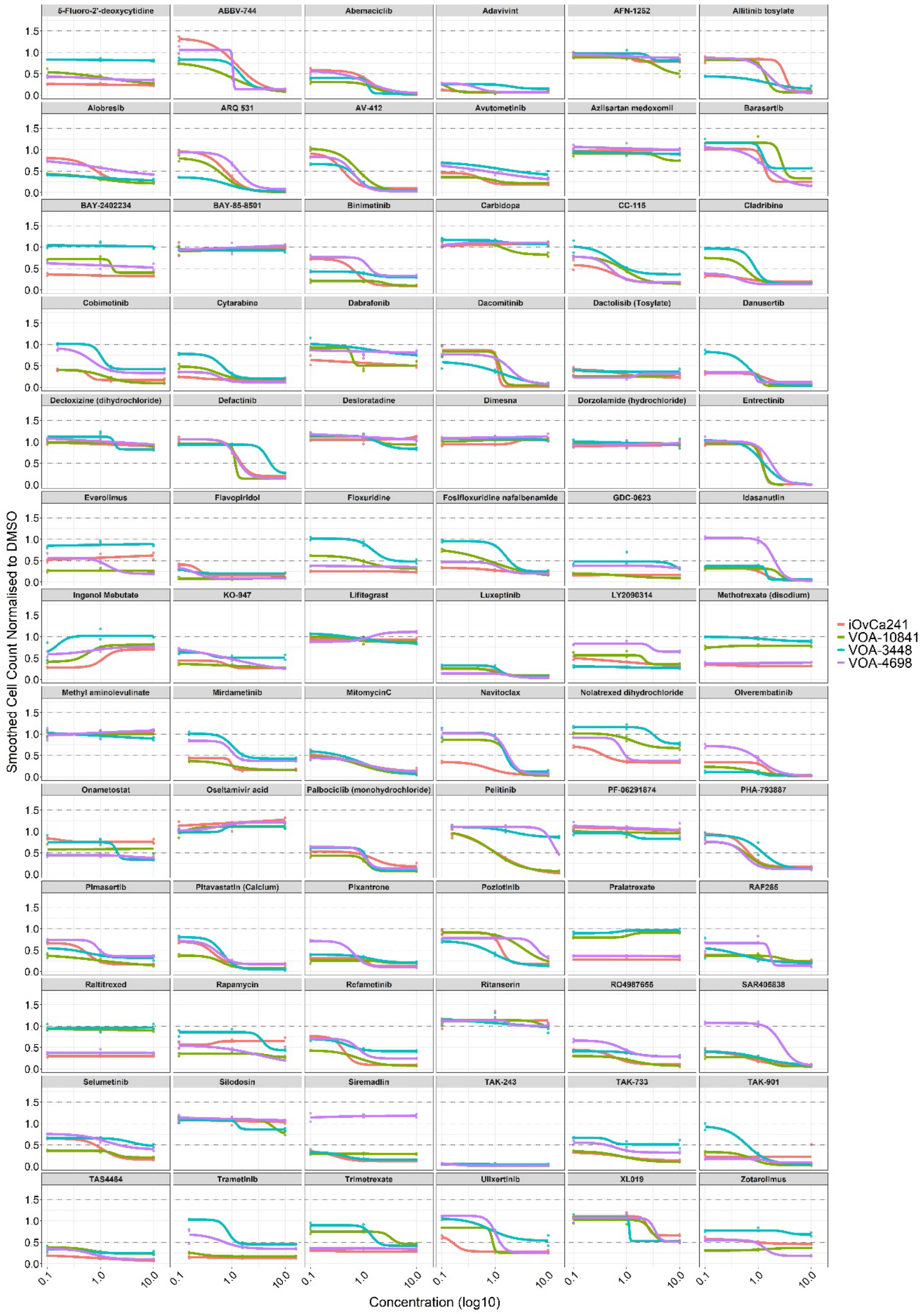


**Supplementary Fig. S2:** Dose response curves of all 78 compounds taken forward for secondary validation across four LGSOC cell lines. Data represents cell counts normalized to DMSO from two technical replicates. Exact chemical formulation of Emetine Dihydrochloride could not be obtained and hence was not validated.
